# Implantable cardioverter‐defibrillator in Brugada syndrome: Long‐term follow‐up

**DOI:** 10.1002/clc.23247

**Published:** 2019-08-22

**Authors:** Ibrahim El‐Battrawy, Gretje Roterberg, Volker Liebe, Uzair Ansari, Siegfried Lang, Xiaobo Zhou, Martin Borggrefe, Ibrahim Akin

**Affiliations:** ^1^ First Department of Medicine, Faculty of Medicine University Medical Centre Mannheim (UMM), University of Heidelberg Mannheim Germany; ^2^ DZHK (German Center for Cardiovascular Research) Partner Site Mannheim Germany

**Keywords:** Brugada, complications, devices, outcome

## Abstract

**Background:**

Brugada syndrome (BrS) is associated with sudden cardiac death (SCD). Although implantable cardioverter‐defibrillator (ICD) implantation is recommended, the long‐term outcomes and follow‐up data with regard to ICD complications have led to controversy.

**Hypothesis:**

In the present study, we described the data assimilated in a total of 11 studies, analyzing the outcome in 747 BrS patients receiving ICD.

**Methods:**

Data were performed and analyzed after a systematic review of literature compiled from a thorough database search (PubMed, Web of Science, Cochrane Library, and Cinahl).

**Results:**

The mean age of patients receiving ICD was (43.1 ± 13.4, 82.5% males, 46.6% spontaneous BrS type I). Around 15.3% of the patients were admitted due to SCD and 10.4% suffered from atrial arrhythmia. Appropriate shocks were documented in 18.1% of the patients over a mean follow‐up period of 82.3 months (47.5‐110.4). The following complications were recorded: lead failure and fracture (5.4%), lead perforation (0.7%), lead dislodgement (1.7%), infection (3.9%), pain (0.4%), subclavian vein thrombosis (0.3%), pericardial effusion (0.1%), endocarditis (0.1%), psychiatric problems (1.5%), pneumothorax (0.7%). Inappropriate shocks were documented in 18.1% of the patients. The management of inappropriate shocks was achieved by pulmonary vein isolation (0.5%), drug treatment with sotalol (1.3%) or sotalol with beta‐blocker (0.3%) and hydroquinidine (0.1%).

**Conclusions:**

ICD therapy in BrS is associated with relevant ICD‐related complications including a substantial risk of inappropriate shocks more frequently in symptomatic BrS patients.

## 1 INTRODUCTION

Type I Brugada syndrome (BrS) is presented by a right bundle branch block (RBBB) and coved ST‐segment elevation in precordial leads (V1‐V3), and its clinical relevance lies in the fact that patients have a pronounced risk to develop malignant tachyarrhythmias.^1^, ^2^ The prevalence of BrS is estimated to be 5/10 000 inhabitants with a higher prevalence in Japan and Philippines as compared to western countries. Not considering accidents, BrS is the leading cause of death in men <40 years old, particularly in countries where the syndrome is endemic. Fever and sodium‐channel blockers could potentially unmask BrS, which have led to an expert consensus advising patients with BrS to avoid these drugs and express caution during clinical states such as fever and infections.^3^

Due to the high risk of sudden cardiac death (SCD), it has been recommended that BrS‐patients with a previous episode of sudden cardiac arrest, or those showing inducibility of a sustained ventricular arrhythmia during an electrophysiological study be treated with an implantable cardioverter‐defibrillator (ICD).^4^ However, ICD is not always feasible or adequate for every patient.

Although alternative treatments including hydroquinine (HQ) treatment and catheter ablation therapy have demonstrated efficacy in recurrent ventricular arrhythmias,^5^ patients who have experienced a prior cardiac arrest or syncopal events secondary to ventricular tachycardia/ventricular fibrillation should undergo ICD implantation.^6^, ^7^ ICD implantation for primary prevention in BrS patients is controversial.^6^, ^8^

The aim of the present study is to observe the long‐term outcome and complication rate of BrS patients, who have received transvenous ICD implantation for primary and secondary prevention.

## 2 METHODS

In this analysis, we included all patients diagnosed with BrS and treated with transvenous ICD implantation 2007 and 2018. A total of 747 BrS patients described in 11 research papers, were recruited for our analysis.

BrS was diagnosed only in the presence of a type 1 Brugada pattern on the electrocardiogram (ECG) (coved type), either at baseline or after the administration of a sodium channel blocking agent. The definition of type 1 ECG pattern was the presence of a terminal r′‐wave with a J‐point elevation of at least 0.2 mV, with a slowly descending ST‐segment followed by a negative T‐wave in ≥1 right precordial lead (V_1_‐V_3_). ECG is placed in the fourth, third, or second intercostal space. Sodium channel blockers were administered intravenously over a 10‐minutes period to unmask the diagnostic ECG pattern of BrS in case of a non‐type 1 ECG pattern at baseline. Programming of ICDs of included studies is summarized in Table S1. Patients were followed annually in a dedicated cardiogenetic outpatient clinic and every 6 to 12 months in the ICD clinic (unless shorter periods of follow‐up were required).

### 2.1 Data collection of different studies

Demographic and clinical data including age at diagnosis, gender, family history of SCD or BrS, symptoms before diagnosis, such as atrial arrhythmias and syncope, results of drug testing, affected genotype, electrophysiological study including ventricular stimulation were followed‐up and evaluated. Baseline ICD‐related data included type of ICD. The indication for ICD implantation was reviewed in different studies, with emphasis on basal ECG characteristics, history of recurrent syncope, inducible VT of VF during programmed ventricular stimulation (PVS), family history of SD, and history of VF or aborted cardiac arrest.

### 2.2 Systematic literature review

**Figure 1:**
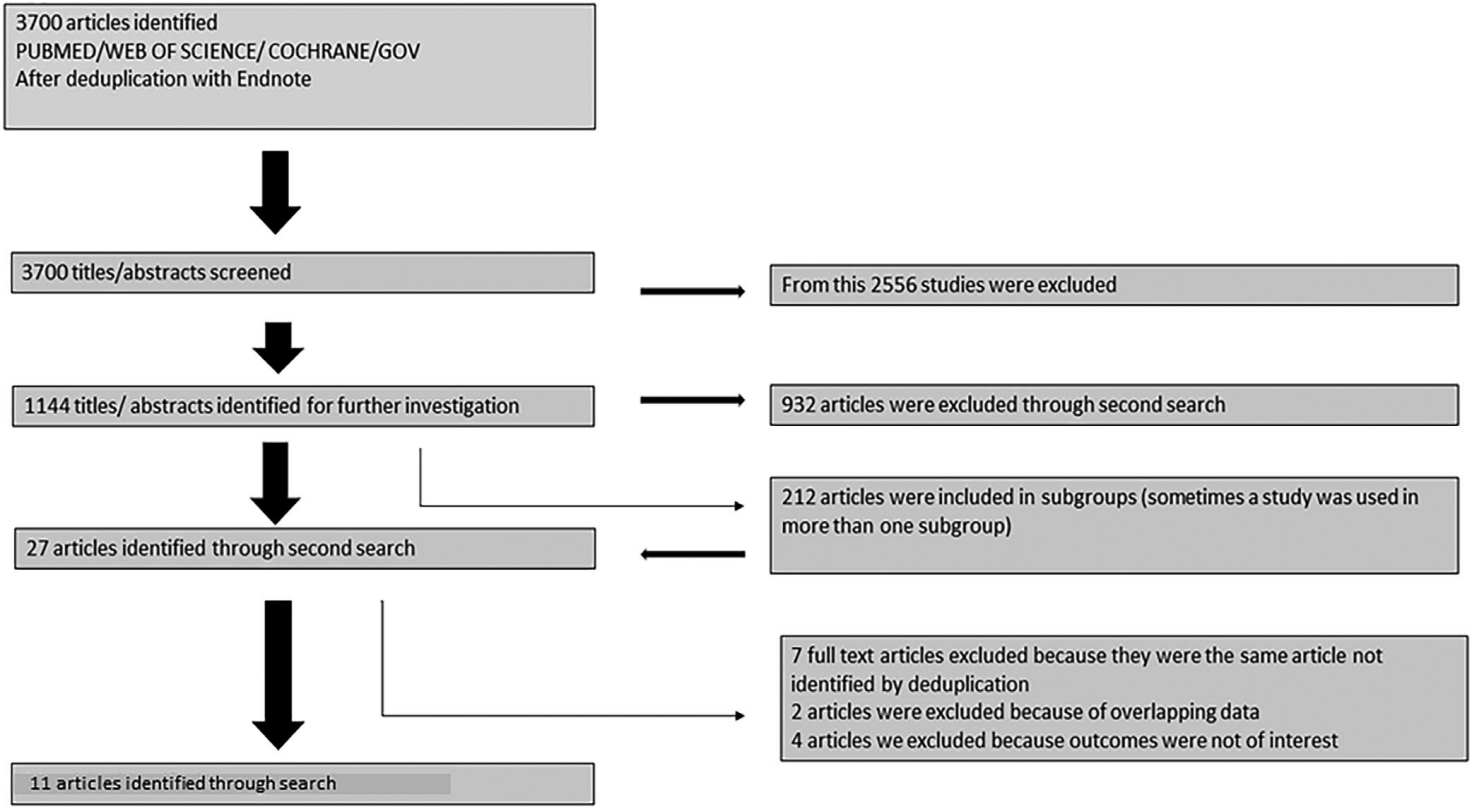
flowchart of recruitment criteria of the present study. Finally, 747 patients were included from 11 studies

A literature search (PubMed, Web of Science, Cochrane Library, and Cinahl) was performed with limits including publication dates to 2018, English language and human subjects. Study selection included the criterion BrS and ICD implantation (Figure 1). Case reports or studies not reporting on outcome of ICD after implantation were excluded.

### 2.3 Statistics

Data are presented as mean ± SD for continuous variables with a normal distribution, median (interquartile range) for continuous variables with a non‐normal distribution, and as frequency (%) for categorical variables. The Kolmogorov‐Smirnov test was used to assess normal distribution. Students *t* test and the Mann‐Whitney *U*‐test were used to compare continuous variables with normal and non‐normal distributions, respectively. The χ² test or Fishers exact test was used to compare categorical variables.

## 3 RESULTS

### 3.1 Demographics

The mean age of patients receiving ICD was 43.1 ± 13.4 with a predominance of males (82.5%). 46.6% of patients showed spontaneous BrS type I and 50.3% demonstrated BrS type I after use of an intravenous sodium channel blocker. Only 21.7% of the patients were asymptomatic. Symptoms were documented as following: 48.3% suffered from recurrent syncope, 15.5% admission due to SCD, and 10.4% atrial arrhythmia, Table S2.

An electrophysiological study (EP) and PVS was performed in 247 patients to study the inducibility of ventricular tachycardia/ventricular fibrillation, and this was documented in 171 patients (69.2% of cases).

### 3.2 ICD‐related complications

**Figure 2:**
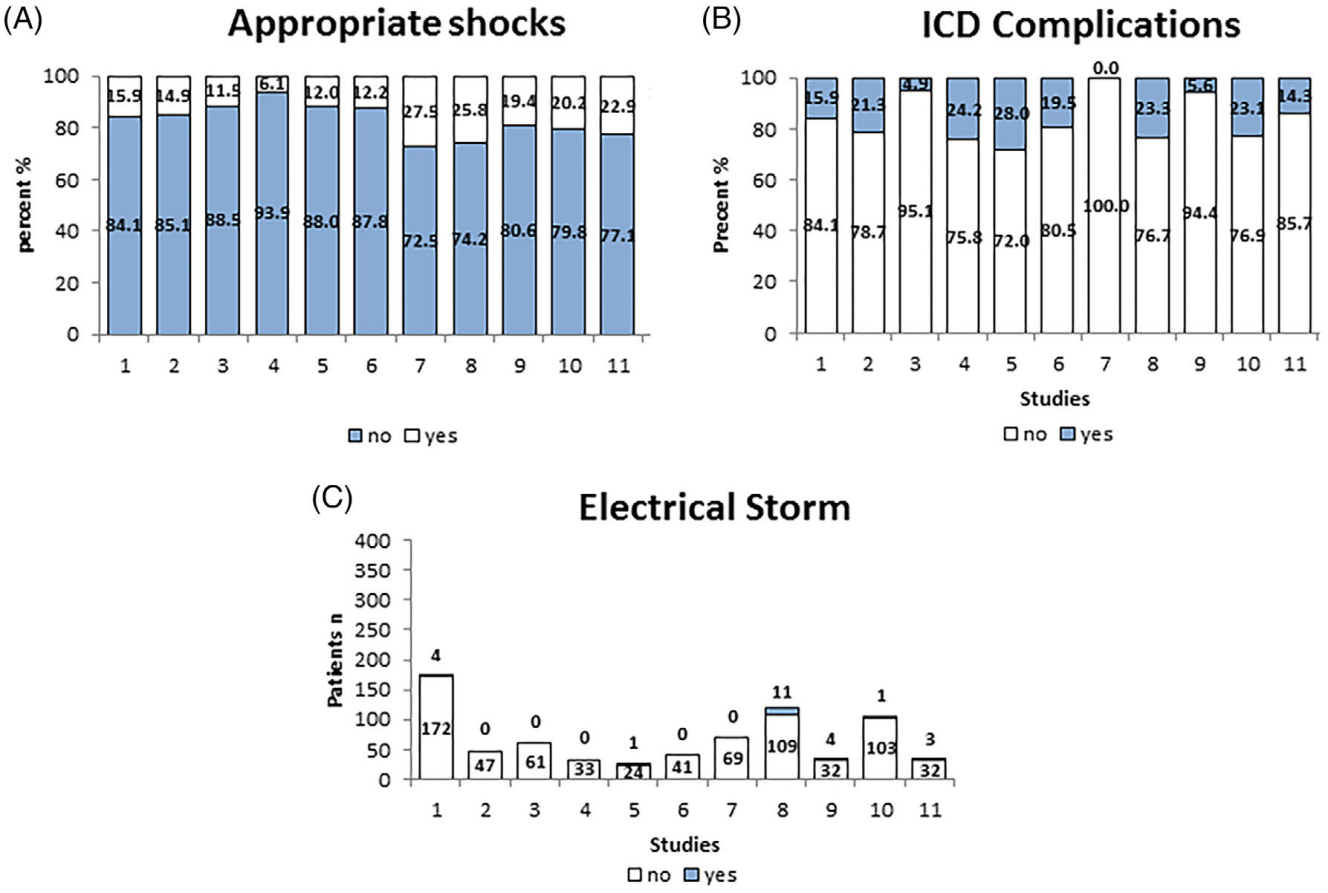
Comparison of different studies showing the outcome of implantable cardioverter‐defibrillator (ICD) in 1201 patients

**Figure 3:**
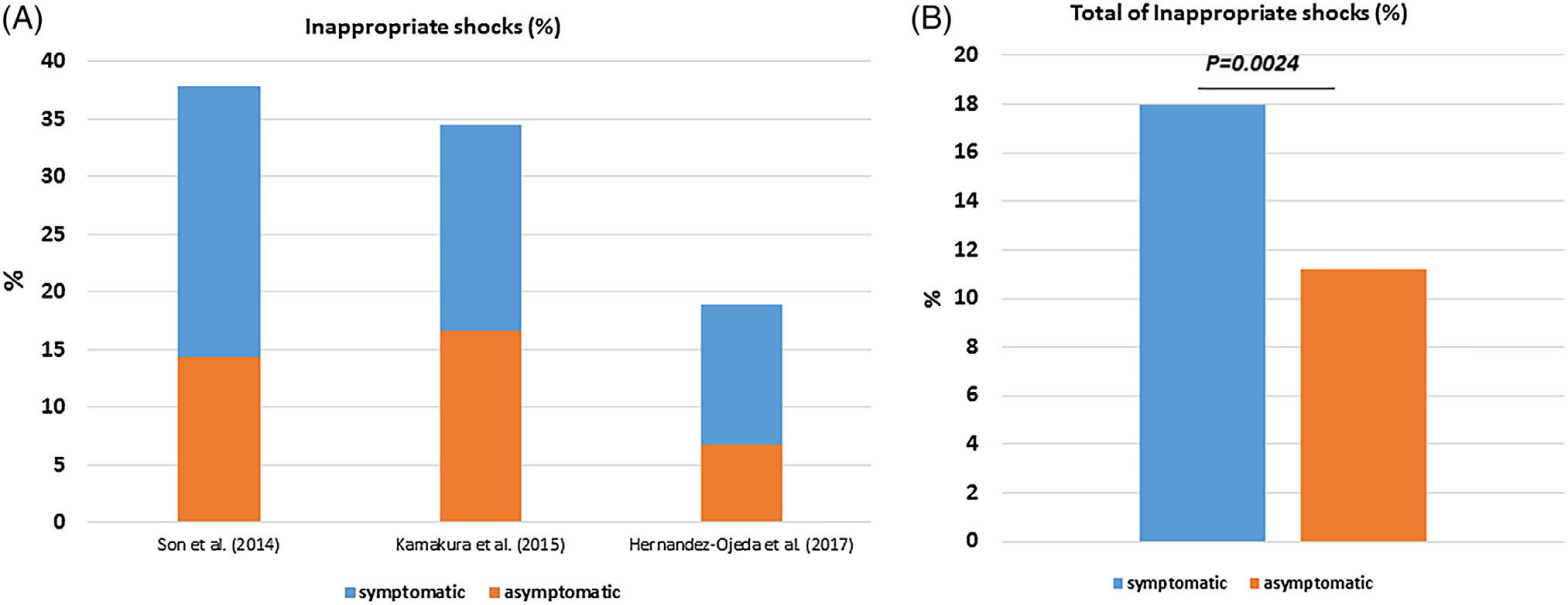
Rate of appropriate and inappropriate implantable cardioverter‐defibrillator **(**ICD) shocks related to the symptomatic state

**Figure 4:**
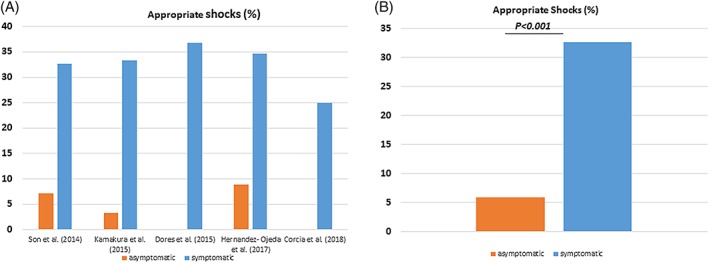
Rate of appropriate and inappropriate implantable cardioverter‐defibrillator (ICD) shocks related to the symptomatic state

The rates of appropriate ICD shocks (18.5%) were similar as compared to inappropriate shocks (18.1%) over a follow‐up interval of 82.3 months (47.5‐110.4). The median time interval to first appropriate shock was 22.3 months, Figure 2A. The complications are listed as follows: lead failure and fracture (5.4%), lead perforation (0.7%), lead dislodgement (1.7%), infection (3.9%), pain (0.4%), subclavian vein thrombosis (0.3%), pericardial effusion (0.1%), endocarditis (0.1%), psychiatric problem (1.5%), pneumothorax (0.7%), Table 1. Inappropriate shocks were attributed to supraventricular arrhythmias (13.7%), noise (3.7%) T‐wave oversensing (2.5%) as well as other causes (0.4%), Table 1 and Figure 2B. Also 3.2% of the patients suffered from an electrical storm. We have compared the data of inappropriate ICD shocks regarding in asymptomatic and symptomatic patients. Asymptomatic BrS patients suffered more significantly from lower rate of inappropriate shocks (Figure 3 A,B). On the other hand, appropriate ICD shocks were significantly more documented in symptomatic patients (Figure 4 A,B).

**Table 1:** ICD‐related complications of 747 BrS patients

Study	Overall	Conte et al^9^	Sarkozy et al^10^	Veltmann et al^11^	Steven et al^12^	Daoulah et al^13^	Miyazakiet al^14^	Son et al^15^	Kamakura et al^16^	Dores et al^17^	Hernandez‐Ojeda et al^18^	Corciaet al 2018
Number of patients	747	176	47	61	33	25	41	69	120	36	104	35
ICD‐complications, n (%)												
ICD‐related complications	143 (19.1)	28 (15.9)	10 (21.3)	13 (21.3)	8 (24.2)	7 (28)	8 (19.5)	0 (0)	28 (23.3)	2 (5.6)	34 (32.7)	5 (14.3)
Lead failure and fracture	40 (5.4)	14 (8.0)	6 (12.8)	2 (3.3)	2 (6.1)	1 (4)	4 (9.8)	0 (0)	10 (8.3)	1 (2.8)	12 (11.5)	3 (8.6)
Lead perforation	5 (0.7)	0 (0)	0 (0)	0 (0)	0 (0)	0 (0)	2 (4.9)	0 (0)	3 (2.5)	0 (0)	0 (0)	0 (0)
Lead dislogement	13 (1.7)	7 (4.0)	0 (0)	0 (0)	0 (0)	1 (4)	1 (2.4)	0 (0)	2 (1.7)	0 (0)	0 (0)	1 (2.9)
Pulse generator migration	5 (0.7)	2 (1.1)	1 (2.1)	0 (0)	0 (0)	0 (0)	0 (0)	0 (0)	0 (0)	0 (0)	1 (1.0)	1 (2.9)
Infection	29 (3.9)	5 (9.1)	0 (0)	1 (1.6)	2 (6.1)	0 (0)	3 (7.3)	0 (0)	10 (8.3)	1 (2.8)	7 (6.7)	0 (0)
Pain	3 (0.4)	0 (0)	0 (0)	0 (0)	0 (0)	0 (0)	0 (0)	0 (0)	3 (2.5)	0 (0)	0 (0)	0 (0)
Subclavian vein thrombosis	2 (0.3)	0 (0)	0 (0)	0 (0)	1 (3.0)	0 (0)	1 (2.4)	0 (0)	0 (0)	0 (0)	0 (0)	0 (0)
Pericardial effusion	1 (0.1)	0 (0)	0 (0)	0 (0)	1 (3.0)	0 (0)	0 (0)	0 (0)	0 (0)	0 (0)	0 (0)	0 (0)
Endocarditis	1 (0.1)	0 (0)	0 (0)	0 (0)	0 (0)	0 (0)	0 (0)	0 (0)	0 (0)	0 (0)	1 (1.0)	0 (0)
Cardiac perforation	1 (0.1)	0 (0)	0 (0)	0 (0)	0 (0)	0 (0)	0 (0)	0 (0)	0 (0)	0 (0)	1 (1.0)	0 (0)
Battery depletion		1.27 ± 0.5 per patient	0 (0)	10 (16.4)	4 (12.1)	0 (0)	0 (0)	0 (0)	0 (0)	0 (0)	73 (70.2)	0 (0)
Psychiatric problem	7 (1.5)	0 (0)	0 (0)	0 (0)	0 (0)	5 (20)	2 (4.9)	0 (0)	0 (0)	0 (0)	0 (0)	0 (0)
High defibrillation threshold	3 (0.4)	0 (0)	2 (4.2)	0 (0)	1 (3.0)	0 (0)	0 (0)	0 (0)	0 (0)	0 (0)	0 (0)	0 (0)
Pneumothorax	5 (0.7)	2 (1.1)	1 (2.1)	0 (0)	0 (0)	0 (0)	0 (0)	0 (0)	0 (0)	0 (0)	2 (1.9)	0 (0)
Shock data												
Inappropriate shocks, n (%)	135 (18.1)	33 (18.8)	17 (36.2)	5 (8.2)	7 (21.1)	3 (12)	10 (24.4)	15 (22.1)	21 (17.5)	8 (22.2)	9 (8.7)	7 (20.0)
Cause for IS, n (%)												
Supraventricular arrhythmia, such as sinus tachycardia, atrial flutter, SVT	102 (13.7)	27 (15.3)	19 (40.4)	1 (1.6)	5 (15.2)	1 (4)	>1 (2.4)	6 (8.7)	32 (26.7)	5 (13.9)	3 (2.9)	2 (5.7)
Sinus tachycardia	27 (3.6)	5 (2.8)	8 (17.0)	0 (0)	0 (0)	1 (4)	0 (0)	Yes	6 (5.0)	5 (13.9)	1 (1.0)	1 (2.9)
SVT	32 (4.3)	0 (0)	6 (12.8)	0 (0)	0 (0)	0 (0)	?	No	26 (21.7)	0 (0)	0 (0)	
Atrial fibrillation	28 (3.7)	15 (8.5)	3 (6.4)	1 (1.6)	5 (15.2)	0 (0)	>1 (2.4)	Yes	0 (0)	0 (0)	2 (1.9)	1 (2.9)
Atrial flutter	3 (0.4)	0 (0)	2 (4.2)	0 (0)	0 (0)	0 (0)	0 (0)	Yes	0 (0)	0 (0)	0 (0)	0 (0)
Noise	28 (3.7)	7 (4.0)	5 (10.6)	0 (0)	0 (0)	1 (4)	0 (0)	2 (2.9)	5 (4.2)	0 (0)	5 (4.8)	3 (8.6)
T‐wave oversensing	19 (2.5)	6 (3.4)	1 (2.1)	4 (6.6)	0 (0)	1 (4)	>1 (2.4)	0 (0)	3 (2.5)	0 (0)	1 (1.0)	2 (5.7)
Abnormal sensing	2 (0.3)	0 (0)	0 (0)	0 (0)	0 (0)	0 (0)	0 (0)	2 (2.9)	0 (0)	0 (0)	0 (0)	0 (0)
NSVT	1 (0.1)	0 (0)	1 (2.1)	0 (0)	0 (0)	0 (0)	0 (0)	0 (0)	0 (0)	0 (0)	0 (0)	0 (0)
Premature ventricular capture beats	2 (0.3)	0 (0)	0 (0)	0 (0)	2 (6.1)	0 (0)	0 (0)	0 (0)	0 (0)	0 (0)	0 (0)	0 (0)
Unknown	1 (0.1)	0 (0)	0 (0)	0 (0)	1 (3.0)	0 (0)	0 (0)	0 (0)	0 (0)	0 (0)	0 (0)	0 (0)
Others	3 (0.4)	0 (0)	0 (0)	0 (0)	0 (0)	0 (0)	0 (0)	0 (0)	3 (2.5)	0 (0)	0 (0)	0 (0)
Management of IS, n (%)												
Pulmonary vein isolation	4 (0.5)	3 (1.7)	0 (0)	0 (0)	0 (0)	0 (0)	0 (0)	0 (0)	0 (0)	0 (0)	0 (0)	1 (2.9)
Drug treatment with sotalol	10 (1.3)	10 (5.7)	0 (0)	0 (0)	0 (0)	0 (0)	0 (0)	0 (0)	0 (0)	0 (0)	0 (0)	0 (0)
Drug treatment with sotalol+betablocker	2 (0.3)	2 (1.1)	0 (0)	0 (0)	0 (0)	0 (0)	0 (0)	0 (0)	0 (0)	0 (0)	0 (0)	0 (0)
Hydroquinidine	1 (0.1)	0 (0)	0 (0)	1 (1.6)	0 (0)	0 (0)	0 (0)	0 (0)	0 (0)	0 (0)	0 (0)	0 (0)
Appropriate shock data, n (%)												
Appropriate shock	138 (18.5)	28 (15.9)	7 (14.9)	7 (11.5)	2 (6.1)	3 (12)	5 (12.2)	19 (27.5)	31 (25.8)	7 (19.4)	21 (20.2)	8 (22.9)
Electrical storm	24 (3.2)	4 (2.3)	0 (0)	0 (0)	0 (0)	≥1 (4)	0 (0)	0 (0)	11 (9.2)	4 (11.1)	1 (1.0)	3 (8.6)
Quinidine treatment	21 (2.8)	2 (1.1)	2 (4.3)	1 (1.6)	0 (0)	1 (4)	0 (0)	0 (0)	2 (1.7)	4 (11.1)	9 (8.7)	0 (0)
Time to appropriate shock, mean (months)	22.3	20.7 ± 25.9	13 (3 days – 4 years)	11 ± 12.6	‐	17 ± 26	‐	16 ± 22	‐	17 (3‐51 months)	48.8 ± 54	7.2
Follow‐up time, mean (months)	83.8 ± 57.3	83.8 ± 57.3	47.5	47.6	94.8 ± 43.2	41.2 ± 17.6	76 (51 to 98)	59 ± 46	102 ± 68	74 ± 40	110.4 ± 60	88 (7‐238)

Abbreviations: ICD, implantable cardioverter defibrillator.; IS, inappropriate shock; n.p., not performed; NSVT, non‐sustained ventricular tachycardia; SCD, sudden cardiac death; SVT, supraventricular tachycardia; VF, ventricular fibrillation; VT, ventricular tachycardia; ‐, no information.

### 3.3 Management of complications

Pulmonary vein isolation was carried out in 0.5% of the patients. About 1.3% received drug treatment with sotalol or sotalol with beta‐blocker (0.3%) and 0.1% received HQ. Electrical storm was documented in 24 patients (3.2%). Therefore, 21 (2.8%) patients were treated with HQ to manage electrical storm, Table 1 and Figure 2C.

## 4 DISCUSSION

We have described the short‐ and long‐term ICD outcomes in 747 BrS patients including 11 defined studies and summarized the following

1. up to 18.5% and 3.2% of all appropriate ICD therapies and electrical storms are documented, respectively with a higher rate in symptomatic BrS patients
2. the incidence of ICD‐related complications, such as inappropriate shocks are common with a rate of up to 18.1% with a higher presence in symptomatic patients
3. management of ICD‐related complications remains challenging. However, use of HQ and ablation strategies may be helpful in managing such complications.

ICD therapy is suggested in survivors of SCD,^6^ however, data have suggested that its use may be associated with significant adverse events.^18^ The primary finding of this systematic analysis is that patients with an ICD implantation for BrS have considerable risk for developing potentially life‐threatening ventricular arrhythmias (18.5%) over the long‐term, as seen in the median follow‐up of 82.3 months. However, inappropriate shocks also occurred in 18.1% of the patients and this was essentially dominated by supraventricular arrhythmias, noise and T‐wave oversensing. The rate of some other ICD‐related complications was also quite high. Our data are consisting with the high rate of inappropriate shocks of other patients treated with ICD implantation, such as in the MADIT II and SCDHEFT trial. Whereas the MADIT (Multicenter Automatic Defibrillator Implantation Trial) II trial has presented a lower rate of inappropriate ICD shocks (11.5%), predominated by atrial fibrillation (44%), supraventricular tachycardia (36%), and abnormal sensing (20%), as compared to our data,^19^ the SCDHEFT trial, recruiting patients with New York Heart Association (NYHA) class II or III heart failure and a left ventricular ejection fraction of 35% or less, reported a comparable rate of inappropriate shocks (17.4%).^20^ Even more in patients with inherited channelopathies including catecholaminergic polymorphic ventricular tachycardia was the rate of inappropriate ICD shocks 24.7%^21^ as compared with 11% in inherited long QT syndrome patients predominated by supraventricular tachycardia.^22^

Overall, there is great variation in the reported rate of appropriate therapy in BrS patients. One of the most significant differences between these studies are the incidence of appropriate therapies in previously asymptomatic individuals. Although several authors report no therapy in this group, during an average follow‐up of 2.3 to 7.3 years,^12^, ^13^, ^23^, ^24^, ^25^ other studies report an overall rate of 4% to 13% after an average follow‐up of 3.2 to 9.3 years.^9^, ^15^, ^26^, ^27^, ^28^ A difference in the study population is the most likely explanation for this observation. Although inappropriate ICD shocks were more documented in symptomatic patients, even more appropriate ICD shocks were also more documented in this group.

BrS and short QT syndrome may be considered similar entities,^29^ thus, comparing patient data from these two groups could prove interesting. Whereas in short QT syndrome, inappropriate therapy is more inherent due to the detection of short‐coupled and prominent T waves (in up to 60% of cases); in BrS, supraventricular arrhythmias are often responsible for the inappropriate shocks. Therefore, careful testing of ICD function and adaptation of sensing levels and decay delays without sacrificing correct arrhythmia detection might be essential for short QT syndrome patient.^30^ On the other hand, in BrS, drug treatment and ablation strategies might be more useful to manage inappropriate shocks.^31^, ^32^

Although only 15.3% of patients were admitted due to aborted out of hospital cardiac arrest and these received ICD implantations for secondary prevention, asymptomatic patients receiving ICDs for primary prevention also suffered from life‐threatening arrhythmias, which were terminated by appropriate ICD shocks. However, further insights in risk stratification strategies are necessary in BrS to avoid ICD‐related complications.

### 4.1 Study limitation

This study provides registry data dominated by retrospective studies and, although the authors clinically evaluated all patients, clinical assessment and treatment algorithm was not uniform and consecutively ICD indications were homogeneous throughout the study. Only* SCN5A* mutations were evaluated in the present analysis; excluding the possibility of mutations in other BrS‐related genes. Also, despite the obvious advantages of our recruited studies, novel therapeutic approaches like ventricular ablation and systematic use of HQ were not evaluated. Finally, the role of subcutaneous ICD was not evaluated in the present study.

## 5 CONCLUSIONS

Regarding a relevant risk of device‐related complications with a higher rate of inappropriate ICD shocks in symptomatic BrS patients special care during regular follow‐up in specialized cardiogenetic centers may allow the reduction in the number of adverse events.

## CONFLICT OF INTEREST

The authors declare no potential conflict of interests.

## Supporting information


**TABLE S1** Programming of implantable cardioverter‐defibrillator (ICDs) reported in included studies

**TABLE S2** Overview of baseline characteristics of Brugada syndrome (BrS) patients receiving implantable cardioverter‐defibrillator (ICD) implantation for primary and secondary prevention
