# Supplementary material for: Implantable cardioverter‐defibrillator in Brugada syndrome: Long‐term follow‐up
Source: Clin Cardiol. 2019 Aug 22;42(10):958–65. doi: 10.1002/clc.23247 (PMC6788474; doi:10.1002/clc.23247)
Supplement: Supplementary file 1 — TABLE S1 Programming of implantable cardioverter‐defibrillator (ICDs) reported in included studies [file CLC-42-958-s001.docx]

**Table S1:** Programming of ICDs

| **Conte et al. 2015** | single VF detection zone and 180 - >200 bmp |
| --- | --- |
| **Sarkozy et al. 2007** | single VF detection zone, 180 b.p.m. (n=19), 190-222 b.p.m. (n=22) |
| **Veltman et al. 2010** | single VF detection zone and a cut-off rate of 222 beats/minute (bpm) |
| **Steven et al. 2011** | VT and VF zones were regularly programmed to high ventricular rate (< 310 ms) |
| **Daoulah et al. 2012** | one VF zone >210 b.p.m. |
| **Miyazakiet al. 2013** | no information |
| **Son et al. 2014** | no information |
| **Kamakura et al. 2015** | decision left to the physician |
| **Dores et al. 2015** | single VF zone with a detection rate of 180-200 bpm |
| **Hernandez- Ojedaet al. 2017** | single VF zone with a detection rate of>200 bpm |
| **Corciaet al. 2018** | single ventricular tachycardia zone at 240 beats/min. |
