# Supplementary material for: Implantable cardioverter‐defibrillator in Brugada syndrome: Long‐term follow‐up
Source: Clin Cardiol. 2019 Aug 22;42(10):958–65. doi: 10.1002/clc.23247 (PMC6788474; doi:10.1002/clc.23247)
Supplement: Supplementary file 2 — TABLE S2 Overview of baseline characteristics of Brugada syndrome (BrS) patients receiving implantable cardioverter‐defibrillator (ICD) implantation for primary and secondary prevention [file CLC-42-958-s002.docx]

| Table S2: Baseline characteristics of Brugada syndrome patients with ICD implantation | | | | | | | | | | | | |
| --- | --- | --- | --- | --- | --- | --- | --- | --- | --- | --- | --- | --- |
| Study | **Overall** | **Conte et al. 2015** | **Sarkozy et al. 2007** | **Veltman et al. 2010** | **Steven et al. 2011** | **Daoulah et al. 2012** | **Miyazakiet al. 2013** | **Son et al. 2014** | **Kamakura et al. 2015** | **Dores et al. 2015** | **Hernandez- Ojedaet al. 2017** | **Corciaet al. 2018** |
| Demographics |  | | | | | | | | | | | |
| Number of patients | 747 | 176 | 47 | 61 | 33 | 25 | 41 | 69 | 120 | 36 | 104 | 35 |
| Age mean±SD | 43.1±13.4 | 43.3±16.8 | 44.5±15 | 42.6±12.9 | 46.4±11.7 | 32±10 | 48±12 | 46.2±13.5 | 46.6±12.2 | 41.7±12 | 46.2± 13 | 13.9± 6.2 |
| Male, n (%) | 616  (82.5) | 118  (67.0) | 35  (74.5) | 41  (67.2) | 30  (90.9) | 25  (100) | 38  (92.7) | 68  (99.0) | 115  (95.8) | 30  (83.3) | 91  (87.5) | 25  (71.4) |
| Symptoms, n (%) |  | | | | | | | | | | | |
| Asymptomatic | 162  (21.7) | 46 (26.1) | 0  (0) | 0  (0) | 0  (0) | 5 (20.0) | 0  (0) | 14 (20.3) | 30  (25) | 19 (52.8) | 45  (43.3) | 3  (8.6) |
| Syncope | 361  (48.3) | 105  (60) | 26 (55.3) | 25  (41) | 23  (70) | 14  (56) | 15 (36.6) | 17 (24.6) | 54  (45.0) | 11  (30.6) | 49  (47.1) | 22  (62.9) |
| SCD | 114  (15.3) | 25 (14.2) | 0  (0) | 7 (11.5) | 3  (9.1) | 6 (24.9) | 9 (22.0) | 38 (55.1) | 0  (0) | 6  (16.7) | 10  (9.6) | 10  (28.6) |
| Atrial arrhythmias | 78  (10.4) | 24 (13.6) | 12 (25.5) | 6  (9.8) | 18 (54.5) | 3 (12.0) | 0  (0) | 0  (0) | 0  (0) | 0  (0) | 0  (0) | 7  (20.0) |
| Family history of SCD | 265  (35.5) | 90 (51.1) | 26 (55.3) | 17 (27.9) | 18 (54.5) | 8 (32.0) | 3  (7.3) | 13 (18.8) | 28  (23.3) | 14 (38.9) | 35  (33.7) | 13  (37.1) |
| ECG Data, n (%) |  | | | | | | | | |  |  |  |
| Drug induced BrS | 376  (50.3) | 139  (79.0) | 41  (87.2) | 34  (55.7) | 16  (48.5) | 4  (16) | 25  (61.0) | 2  (2.9) | 35  (29.2) | 11  (30.6) | 39  (37.5) | 30  (85.7) |
| Spontaneous BrS Type I | 348  (46.6) | 37 (21.0) | 6 (12.8) | 26 (44.3) | 18 (54.5) | 22 (88.0) | 15 (36.6) | 44 (63.7) | 85  (70.8) | 25 (69.4) | 65  (62.5) | 5  (14.3) |
| ICD Implantation, n (%) |  | | | | | | | | |  |  |  |
| yes | 747  (100) | 176 (100) | 47 (100) | 61 (100) | 33 (100) | 25 (100) | 41  (100) | 69 (100) | 120  (100) | 36  (100) | 104  (100) | 35  (100) |
| ICD Complications, n (%) |  | | | | | | | | |  |  |  |
| yes | 143  (19.1) | 28 (15.9) | 10 (21.3) | 13 (21.3) | 8 (42.2) | 2  (8.0) | 8 (19.5) | 0  (0) | 28  (32.3) | 2  (5.6) | 34  (32.7) | 5  (14.3) |
| Adequate shocks, n (%) | 138  (18.5) | 28 (15.9) | 7 (15.0) | 7 (11.5) | 2  (6.1) | 3 (12.0) | 5 (12.2) | 19 (27.5) | 31  (25.8) | 7  (19.4) | 21  (20.2) | 8  (22.9) |
| EP study data, n (%) | | | | | | | | | | | | |
| EP study | 247  (33.1) | 165 (93.8) | 46 (98.0) | 52 (85.2) | 25 (75.8) | 18 (72.0) | 35 (85.4) | n.p. | 100  (83.3) | 26 (72.2) | 95  (91.3) | 26  (74.3) |
| Induction of FV or VT | 171  (69.2) | 72 (43.6) | 14 (30.4) | 40 (76.9) | 14 (56.0) | 18 (100) | 33 (94.3) | n.p. | 80  (80) | 22 (84.6) | 65  (68.4) | 4  (15.4) |
| Genetic screening, n (%) |  | | | | | | | | |  |  |  |
| SCN5a | 80  (10.7) | 23  (21.9) | n.p. | 14  (23) | n.p | n.p. | n.p. | n.p. | 20/80  (25) | n.p. | 16/68 (23.5) | 6/19  (31.6) |
| Follow- up time, mean (months) | 82.3 (47.5-110.4) | 83.8 | 47.5 | 47.6 | 94.8 | 41.2 | 76 | 59 | 102 | 74 | 110.4 | 88 |
| ICD=implantable cardioverter defibrillator, - = no information, n.p.= not performed, VT= ventricular tachycardia, VF=ventricular fibrillation, SCD=sudden cardiac death | | | | | | | | | | | | |
